# Artificial intelligence in drug repurposing for rare diseases: a mini-review

**DOI:** 10.3389/fmed.2024.1404338

**Published:** 2024-05-22

**Authors:** Lucas Cortial, Vincent Montero, Sébastien Tourlet, Joanie Del Bano, Olivier Blin

**Affiliations:** ^1^OrphanDEV FCRIN Reference Network, Aix Marseille Univ, APHM, INSERM, Inst Neurosci Syst, CHU Timone, Marseille, France; ^2^Thelonius Mind, Marseille, France; ^3^MyDisease2Ez, Fresnes, France

**Keywords:** artificial intelligence, machine learning, deep learning, drug repurposing, rare diseases

## Abstract

Drug repurposing, the process of identifying new uses for existing drugs beyond their original indications, offers significant advantages in terms of reduced development time and costs, particularly in addressing unmet medical needs in rare diseases. Artificial intelligence (AI) has emerged as a transformative force in healthcare, and by leveraging AI technologies, researchers aim to overcome some of the challenges associated with rare diseases. This review presents concrete case studies, as well as pre-existing platforms, initiatives, and companies that demonstrate the application of AI for drug repurposing in rare diseases. Despite representing a modest part of the literature compared to other diseases such as COVID-19 or cancer, the growing interest, and investment in AI for drug repurposing in rare diseases underscore its potential to accelerate treatment availability for patients with unmet medical needs.

## 1 Introduction

Drug repurposing is defined as the process of identifying a new use for an existing drug or active substance in an indication outside the scope of the original indication. It represents important advantages, including reduced development time and costs and potentially large societal healthcare cost savings. Drug repurposing represents a significant innovative option for therapies for rare diseases. Indeed, among the over 7,000 identified rare diseases affecting 4% of the worldwide population, less than 6% of them have an approved treatment option (1, 2). Because of the factors inherent to rare diseases (i.e., limited patient populations, disease complexity, and lack of understanding), the development of new therapies is challenging. In rare diseases, the drug repurposing process results in a reduction of the risk of drug development, and, ultimately, in the acceleration of the available treatment options for patients (3).

Concurrently, artificial intelligence (AI)—including techniques such as machine learning (ML), deep learning (DL), or even natural language processing (NLP) (4)^1^, (5)^2^—has emerged as a transformative force in the domain of healthcare, offering unprecedented opportunities for drug discovery, virtual clinical consultation, disease diagnosis, prognosis, medication management, health monitoring, genomic medicine, or even patient care (6, 7). In the context of rare diseases, where data are often scarce and heterogenous, the ability of AI technologies to integrate and analyze data from diverse sources, including electronic health records, genomic data, biomedical literature, or patient registries, can be used to overcome the challenges associated with rare diseases (8).

Ultimately, the synergy between AI and drug repurposing has therefore been defined as holding promises for addressing the unmet medical needs in rare diseases (9, 10). In this review, we provide an overview of the current landscape of AI drug repurposing in rare diseases, showcasing concrete case studies from the literature and highlighting existing platforms, libraries, funded initiatives, and companies at the forefront of this emerging field.

## 2 Methods

### 2.1 Literature research

Literature research was performed on the MEDLINE database, using a combination of MeSH terms designed to maximize the retrieval of relevant results aligned with the objectives of the review. The following combination was used: *[(artificial intelligence) OR (machine learning) OR (deep learning) OR (natural language processing)] AND [(drug repurposing) OR (drug repositioning)]*.

All the literature references were then extracted (11 March 2024) under the PubMed format. This format, which replaced the RIS format, allows saving citations as a text or .nbib file matching the MEDLINE format (11).

For further categorization of the retrieved literature, particularly regarding the diseases addressed by the references, we established a list of keywords (as presented in Table 1) for each disease detected in the references. These keywords were selected based on (1) their relevance to the disease category and on (2) their ability to categorize a high proportion of relevant references. Subsequently, a custom Python script was used to match these specified keywords with the abstracts of the literature references retrieved from the initial search. This approach allowed us to categorize the references according to the diseases they address.

**Table 1:** Disease categorization mapping keywords.

Cancer	Cancer, oncology, oncologic, anti-cancer, anticancer, carcinoma
Covid	COVID-19, COVID, SARS-CoV-2, coronavirus
Rare diseases	Rare diseases; orphan; orphan diseases; orphan drugs; RDs; rare genetic disorder
Neurodegenerative diseases	Alzheimer, Alzheimer’s disease, Parkinson, Parkinson’s disease, aging, and neurodegenerative
Neurological diseases	Neurological disorders, neurological, schizophrenia, anxiety, depression, psychiatric, autism, and epilepsy
Renal diseases	Renal and kidney
Liver diseases	Liver and liver disease
Pain	Pain, dolor, analgesic, and antalgic
Inflammatory diseases	Inflammatory diseases and inflammatory
Tropical diseases	Tropical diseases and tropical
Cardiovascular diseases	Cardiovascular disease and cardiovascular
Respiratory diseases	Respiratory disease, respiratory, pulmonary, and lung
Immunologic diseases	Immune, immunology, autoimmune disease, allergic, and allergic disorders
Skin diseases	Skin disease and psoriasis
Infectious disease	Infectious disease, infectious, virus, bacteria, viruses, viral, and bacterial
Misuse disorders	Abuse, use disorder, and drug addiction
Hematology	Hematology, hematology, and hematological
Diabetes	Diabetes, diabetic, and anti-diabetic

### 2.2 Exploring AI-driven initiatives: beyond scientific literature

In tandem with our literature review, we investigated the existent initiatives leveraging AI tools for drug repurposing in rare diseases. This exploration encompassed two integrated and more general platforms: Google and LinkedIn. We selected all the existent initiatives (companies, open-source or private platforms, libraries, etc.) that explicitly mention using AI tools for drug repurposing in rare diseases.

## 3 Results

**Figure 1:**
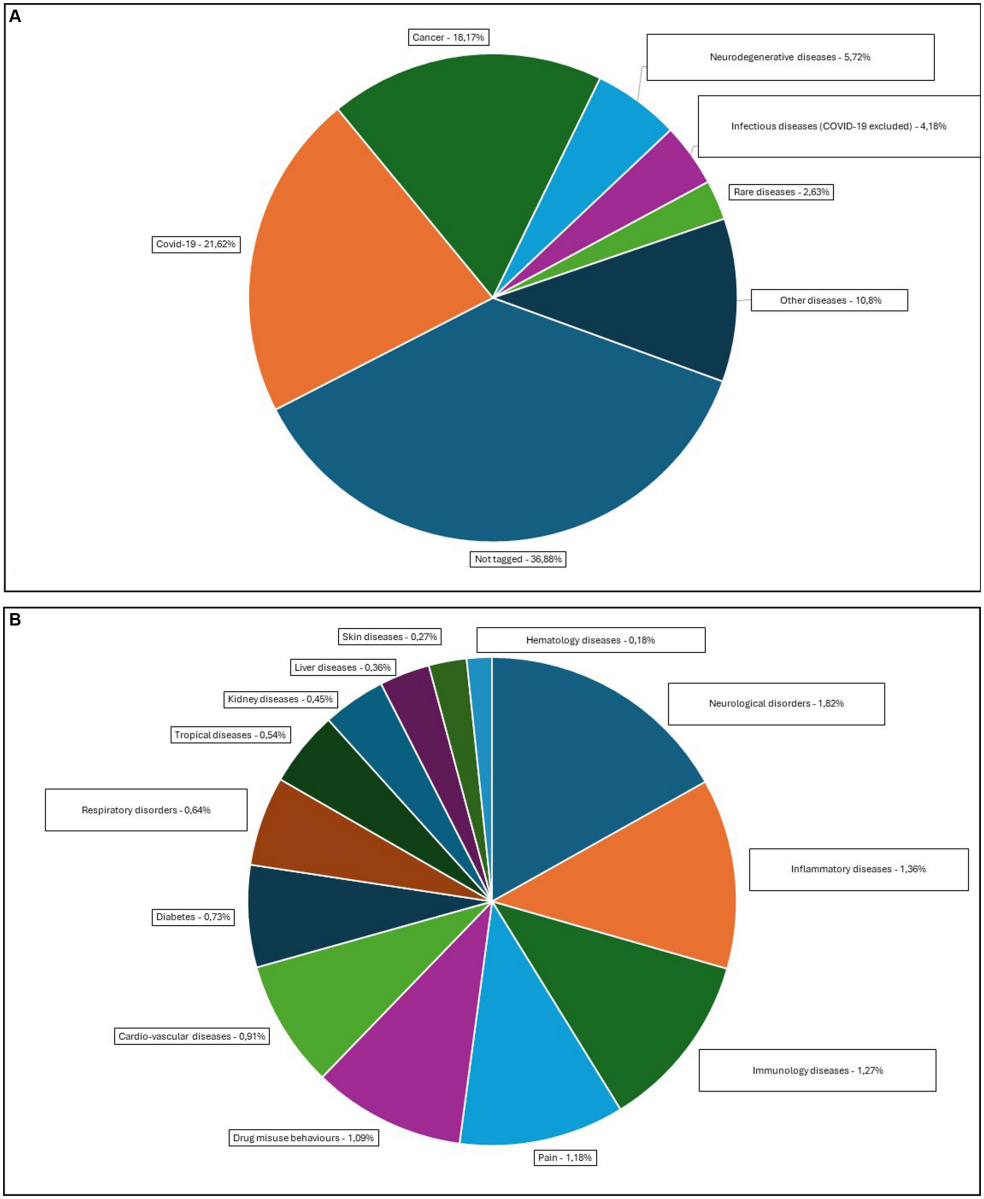
Overview of the diseases targeted by AI applications in drug repurposing. **(A)** Overview of the diseases representing more than 2% of the retrieved references: COVID-19, cancer, neurodegenerative diseases, infectious diseases, rare diseases, and other diseases (*diseases representing less than 2% of the retrieved references*) and not tagged. **(B)** Overview of the diseases representing less than 2% of the retrieved references: neurological disorders, inflammatory diseases, immunology diseases, pain, drug misuse behaviors, cardiovascular diseases, diabetes, respiratory disorders, tropical diseases, kidney diseases, liver diseases, and skin diseases.

Through the literature research, we have been able to collect a total of 1,090 bibliographic references relating to the use of AI for drug repurposing. Among them, we were able to categorize a total of 677 references according to the diseases they address (62.11%). A total of 18 disease categories were identified (Table 1). The distribution of the different categories is presented in Figures 1A,B.

The most common disease categories in which AI has been applied and defined as a promising asset for drug repurposing are COVID-19 (21.62%), cancer (18.17%), neurodegenerative diseases, Parkinson’s and Alzheimer’s disease being the most targeted diseases (5.72%), and infectious diseases, with COVID-19 being excluded (4.18%). Rare diseases are ranked as the fifth most cited diseases, accounting for 2.63% of the literature references.

### 3.1 Concrete applications of AI in drug repurposing for rare diseases

The categorization of the literature references allowed us to retrieve a total of 28 references related to rare diseases. Among these references, we identified six references describing the identification of candidates for drug repurposing in various rare diseases using different AI technologies. The other 22 articles that are not described in this review were journal articles, reviews, or research supports that did not describe concrete applications of AI in drug repurposing.

- In 2019, Ekins et al. (12) described the application of ML models for 180 approved drugs to identify potential drug repurposing for the treatment of Pitt–Hopkins syndrome (PTHS). Ekins et al. (12) first performed a high-throughput screen (HTS) of the Prestwick chemical library that allowed the identification of 55 inhibitors of K_v_7.1 and 93 inhibitors of Na_v_1.8 as leads for the treatment of PTHS. They then aimed to compare their results to that of the Bayesian ML model in Assay Central. This model allowed the prediction of 35 K_v_7.1 and 64 Na_v_1.8, suggesting that the combination of HTS and ML represents a strength for drug repurposing in rare diseases (12).
- In 2020, Sosa et al. (13) implemented a literature-based graph embedding method for drug repurposing that explicitly models the confidence relationship in the Global Network of Biomedical Relationships (GNBRs) based on their evidence in the literature. GNBR is a heterogeneous knowledge graph (KG) that contains the synthesis and summary of drugs, genes/proteins, and disease relationships described by 28.6 million PubMed references. By matching the information contained in the GNBR with rare disease information extracted from Orphanet—including MeSH, OMIM, and UMLS ID—Sosa et al. (13) retrieved 2,779 rare diseases. The method developed allows to compile a score for the top 30 drug repurposing candidates, in which the validity of the prediction is categorized into six categories: (1) published treatment, meaning that there is literature evidence indicating the use of the drug to treat human subjects; (2) symptom management, meaning that the drug has been used to address symptoms of the disease; (3) co-morbidity treatment, meaning that the drug treats a comorbid or closely related disease; (4) potentially feasible treatment, meaning that there is pre-clinical and/or biologically tractable evidence for the drug targeting the rare disease; (5) possible contraindication, meaning that the drug may produce a physiological effect opposite of the desired one; and (6) unknown/no-effect, where there is little or no literature evidence to support the drug-disease combination. In addition to the correct identification of treatments that have been published in the literature but not retrieved in the GNBR (i.e., cortisone for myelodysplastic syndrome or famotidine for leishmaniasis), the method demonstrates an additional inductive capacity of the model developed. Indeed, two drug candidates were identified as promising candidates for further clinical study: Trifluoperazine as a treatment for Wilms tumor and mTOR inhibitors (Everolimus) as a treatment for sarcoidosis (13).
- In 2021, Esmail and Danter (14) were a to identify new potential therapeutic options for the treatment of metachromatic leukodystrophy (MLD) using artificially generated whole-brain organoid (aiWBO) simulations of MLD. These simulations were generated using the NEUBOrg (artificially induced whole-brain organoid platform), a stem cell simulation platform with a literature-validated hybrid deep-ML system with elements of fully connected recurrent neural networks, cognitive maps, support vector machines, and evolutionary systems. Applied to MLD, NEUBOrg was used to simulate a whole-brain organoid affected by arylsulfatase A (ARSA) deficiency, resulting in the outcome of the disease modeling simulations (aiWBO-MLD). MLD drug targets were identified based on AI WBO-MLD disease profile simulations, and a list of drugs was generated. Specifically, a list of 861 single-drug and double-drug combinations was generated and evaluated by the aiWBO-MLD simulations. The drugs were then ranked according to their effects compared to placebo, and the top 12 options were obtained. All of the options were double drug combinations—regorafenib + olaparib; pembrolizumab + lenvatinib; sunitinib + lenvatinib; lenvatinib + capmatinib; rapamycin + lenvatinib; regorafenib + lenvatinib; regorafenib + calpain inhibitor; sunitinib + nutlin3; sunitinib + olaparib; olaparib + abemaciclib; palbociclib + olaparib; and ribociclib + olaparib—and display an ameliorating effect on the nine factors of MLD disease (14).
- In 2022, Cong et al. (15) presented a double-sided approach to drug repurposing by combining the gene expression responses of cell lines caused by diseases with data on drug-induced changes in expression. The authors obtained gene expression datasets from 262 patients and 268 healthy human samples associated with 31 diseases directly from the National Center for Biotechnology Information’s (NCBI) Gene Expression Omnibus (GEO). The 31 diseases were then clustered into several classified groups according to disease similarity, and three diseases were selected—according to the *k*-means method—for further analysis: inclusion body myositis (IBM), polymyositis (PM), and dermatomyositis (DM). Using L1000CDS^2^, a search engine allowing the prioritization of small molecules (SMs) and drugs to either reverse or mimic observed changes in gene expression, different drugs were identified as able to reverse the expression pattern changes for each of the three diseases: 10 drugs corresponding to IMB and PM, including gemcitabine, tosedostat, wortmannin or alvocidib; and 10 drugs corresponding to PM and DM, including selumetinib, wiskostatin or perhexiline maleate; and 14 drugs for all diseases, including salermide, wortmannin or reserpine (15).
- In 2022, Foksinka et al. (16) demonstrated that mediKanren, an AI reasoning tool that uses KGs for describing relationships between biomedical concepts, aids in treatment identification. Specifically, in cases where upregulating or downregulating a gene could be beneficial, mediKanren helps identify drugs or compounds that may accomplish the desired outcome. Coupled with literature research, the results can be ranked according to the strength of evidence for the therapeutics, and the top drug candidates are prioritized based on accessibility, bioavailability, and safety. As a case in point, this allowed to identify two FDA-approved drugs, celecoxib and levocarnitine, as respective potential therapeutics for the treatment of patients presenting *RHOBTB2* and *TMLHE* variants (16).
- In 2023, Zhu et al. (17) constructed RDKG-115, a rare disease KG involving 115RDs, gleaned from high-quality literature and 4 biomedical datasets: (1) DRKG, a comprehensive KG for drug repurposing, (2) Pathway Commons, an integrated resource providing information on biological pathways and molecular integrations (gene, SMs, and drug entities), (3) PharmKG, a benchmark KG for biomedical mining (gene, drug, and disease entities), and (4) PMapp, a knowledge base application platform for precision medicine. Five types of entities were retrieved in these datasets: disease (DI), drug (DR), gene and gene product (GP), phenotype (PH), and SM data. RDKG-115 was then constructed through entity linking, relation linking (e.g., DI–DI or DR–GP relationships), and entity frequency screening. For the 115 RDs, Zhu et al. (17) were able to analyze the DI-DR and DI-SM relationships to identify the potential drugs and SMs repurposing. Compared to the drug-related entity pairs gathered from the 932 clinical trial records extracted from ClinicalTrials.gov (CTG) for 115 RDs, the analysis method allowed for the retrieval of approximately half of the clinical trials (48.3%).

Focusing on the most studied rare diseases in CTG, multiple sclerosis (MS), RDKG-115 allowed us to demonstrate that melatonin and amyloid-β were the two drugs exhibiting the most promising association with MS. (17)

### 3.2 Existent initiatives for drug repurposing in rare diseases

In addition to the concrete case studies demonstrating the application of AI technologies for drug repurposing in rare diseases, we can also identify several platforms and libraries, funded initiatives, and companies dedicated to drug repurposing in rare diseases using AI.

#### 3.2.1 Platforms and libraries

##### 3.2.1.1 Every Cure platform

Every Cure^3^ is a platform funded by the Advanced Research Projects Agency for Health (ARPA-H) aiming to allow the development of a comprehensive, open-source database of drug repurposing opportunities and to launch clinical trials with the most promising treatments (18). This platform obtains, integrates, and analyses multiple data sources (PubMed, clinicaltrials.gov, medical record data, public data repositories, and drug databases), utilizes NLP and applies an ML algorithm to identify the most promising drug repurposing opportunities. Every Cure approach has previously allowed for the identification of drug repurposing opportunities for Castleman disease (adalimumab), COVID-19 (dexamethasone and tocilizumab), and angiosarcoma (pembrolizumab).

##### 3.2.1.2 REPO4EU

REPO4EU^4^ is a European Union-funded platform for validated precision drug repurposing open to stakeholders for information, multimedia training, matchmaking, and cooperation (19). REPO4EU proposes a European and global platform for mechanism-based drug repurposing, redefining diseases by applying advanced bioinformatics and AI to real-world big data.

##### 3.2.1.3 Open Targets

This innovative, large-scale, multi-year, public-private partnership uses human genetics and genomics data for systematic drug target identification and prioritization (20).^5^ The OpenTargets Platform integrates public domain data to enable target identification and prioritization, whereas OpenTargets Genetics identifies targets based on Genome-Wide Association Studies (GWAS) and functional genomics.

Open Targets is a consortium of partner institutions, including major pharmaceutical companies Bristol Myers Squibb, Genentech, GSK, Pfizer, Sanofi, and Wellcome Sanger Institute, but also the European Bioinformatics Institute (MBL-EBI).

##### 3.2.1.4 Broad Drug Repurposing Hub

The Drug Repurposing Hub is an open-access drug library and information resource repository of more than 6,000 compounds, aiming to accelerate drug development and drug repurposing (21).^6^

The Drug Repurposing Hub has been developed through (1) the identification of compounds, (2) the comprehensive annotation of their known activities and clinical indications, and (3) the experimental confirmation of the drug’s identity and purity. Ultimately, the Hub proposes to the scientific community a resource containing annotations—name, chemical structure, clinical trial status, mechanism of action, protein targets, disease areas, approved indications, external links, purity, and vendor ID—of more than 6,000 drugs reaching phases 1–3 in clinical development (22).

##### 3.2.1.5 REMEDi4ALL

Launched in September 2022, REMEDi4ALL is a 5-year EU-funded research initiative aiming to drive forward the repurposing of medicines in Europe (23).^7^ To this end, REMEDi4ALL will build a state-of-the-art platform to provide expertise and services across the complete value chain for patient-centric medicine repurposing, assemble advanced *in silico* tools for ML and AI, create a global community of practice, train and educate the next generation of stakeholders and favor dialogue and debate to advance policy and fair access to repurposed medicines across the EU. The therapeutic areas that are being covered by the project are pancreatic cancer, COVID-19, rare diseases, and ultra-rare diseases.

#### 3.2.2 Funded initiatives

##### 3.2.2.1 DREAMS

The European Commission has invested €8 million into the Drug REpurposing and Artificial intelligence for Muscular disorders (DREAMS) consortium. Announced on 29 November 2023, this 5-year project has the objective of crafting treatment for five rare neuromuscular diseases—Duchenne muscular dystrophy, centronuclear myopathy, Emery–Dreifuss muscular dystrophy, Pompe disease, and Danon disease—through a groundbreaking methodology combining AI, stem cells, and pharmacological screening (24). This consortium unites a skilled and complementary consortium of European partners, including I-Stem (France), Kantify (Belgium), The Institute of Myology (France), Center for Neuroscience and Cell Biology (Portugal), The Technion (Israel), Samsara Therapeutics (United Kingdom), Assistance Publique—Hopitaux de Paris (France), AFM-Telethon (France), and Zabala Innovation (Spain).

#### 3.2.3 Companies

##### 3.2.3.1 HealX

The UK-based company leverages AI technology to identify promising drugs and compounds already approved or in clinical development that could be repurposed to treat rare diseases (25).^8^ Through its platform, HealNet and HealX use ML methods to extract disease knowledge from a variety of published sources and predict which drugs and combination therapies are most likely to succeed in the clinic. Since its inception, the company has entered a number of collaborative partnerships with academics, patients, and industry groups aimed at discovering new treatments for a range of rare conditions, such as Friedreich’s ataxia or muscular dystrophy.

At the time of writing (17 March 2024), the HealX pipeline focuses on 15 conditions including oncology disorders (neurofibromatosis type 1: cutaneous neurofibroma and plexiform neurofibroma), neuro-developmental disorders (Fragile X syndrome and Angelman Syndrome), renal/liver disorders (autosomal dominant/recessive polycystic kidney disease and autosomal dominant polycystic liver disease), neuromuscular disorders (myotonic dystrophy type-1, spinocerebellar ataxia and pseudoachondroplasia), ocular disorders (leber hereditary optic neuropathy and autosomal dominant optic atrophy) and other exploratory programs (Alport syndrome, alpha-1 antitrypsin deficiency, facioscapulohumeral muscular dystrophy and pitt hopkins). Noteworthy, HealX received (1) Investigational New Drug (IND) approval from the FDA for the phase 2a clinical trial of their AI-discovered treatment, Sulindac, a non-steroidal anti-inflammatory drug, for the treatment of fragile X syndrome in 2021, as well as an Orphan Drug Designation (ODD) in Europe and in the US for the same compound, and (2) a US ODD for their AI-discovered treatment, Nitroxoline, an antibiotic, for the treatment of Neurofibromatosis type 1 in 2023.

##### 3.2.3.2 Biovista

Biovista applies its systematic discovery AI platform, Project Prodigy, to develop a pipeline of repositioned drug candidates in different disease areas, including neurodegenerative diseases, epilepsy, oncology, and orphan diseases (26).^9^ The platform analyzes massive data resources and identifies non-obvious, mechanism of action-based associations between compounds, molecular targets, and diseases. Biovista partners with biopharmaceutical and biotechnology companies (Astellas, Pfizer, Hewlett Packard Enterprise, Novartis, Dart Therapeutics, etc.), as well as public and private organizations and academic institutes.

## 4 Discussion

Through this mini-review, we have been able to characterize the proportion represented by rare diseases in the literature describing the application of AI for drug repurposing. Compared to the other diseases categorized (e.g., COVID-19, cancer, or neurodegenerative diseases), rare diseases only represent 2.63% of the overall literature, which can also be found regarding the broader use of AI in rare diseases, where, compared to other application domains (e.g., disease diagnosis, gene identification, and drug discovery), drug repurposing is also a relatively less prominent area of focus (27, 28). This low representation of rare diseases in the literature on AI-driven drug repurposing may be attributed to several factors, including the complexity of rare diseases, the small number of patients affected by rare diseases, the limited availability of data, the variability in symptoms, the genetic diversity among patients, or even the lack of funding priorities in rare diseases, making research in rare diseases not only challenging but also economically unappealing.

However, different applications have demonstrated that AI in drug repurposing is promising and that it will benefit patients suffering from rare diseases. Indeed, six concrete applications illustrated diverse strategies regarding drug repurposing in rare diseases: Ekins et al. combined HTS and ML to identify potential treatments for PTHS; Sosa et al. (13) used literature-based graph embedding to categorize drug repurposing candidates in various rare diseases. Esmail and Danter (14) generated artificial brain organoids to identify promising drug combinations for the treatment of MLD. Cong et al. used gene expression data to match diseases with potential drug candidates, while Foksinka et al. (16) used AI reasoning tools to identify therapeutics based on gene regulation, and, finally, Zhu et al. (17) constructed a rare disease KG to identify potential drugs and SMs for repurposing in various rare diseases. This momentum surrounding AI-driven drug repurposing in rare diseases is also reflected in the various current initiatives transcending core research. Scientists have developed algorithms, platforms, and libraries to inform and assist the scientific community. Companies have made it their business to identify and obtain regulatory approvals for promising drugs, while public bodies and big pharma’s involvement is already being manifested.

The landscape of AI-driven drug repurposing in rare diseases presents an intriguing paradox. We observe a scarcity of research, companies, and funding dedicated to leveraging AI for drug repurposing in rare diseases. Given the substantial financial incentives for pharmaceutical companies to pursue drug repurposing compared to the development of new drugs from scratch and given the current AI tools rapidly expanding across various sectors, including health, one can wonder why it is so rare, especially when patients with rare diseases are often treated with off-label drugs. Is the complexity of rare diseases truly hindering drug repurposing efforts? Are current AI tools not yet capable of identifying potential drug repurposing opportunities for rare diseases? Or perhaps these efforts are underway but kept low-profile by industry players to safeguard intellectual property, considering the intricate nature of AI’s intellectual property landscape? Another aspect worth considering is the question of profitability in treating rare diseases. Could concerns about the financial viability of treatments for rare diseases be a factor in the relative lack of investment in AI-driven drug repurposing, notably because it requires expertise that is scarce on the market and therefore inevitably expensive?

In light of these reflections, it would not be surprising to witness a surge of small biotech or tech companies emerging in the coming year, dedicated to the repurposing of therapies, whether polypharmacological compounds or generics, for rare diseases using AI-driven approaches. Such initiatives could hold tremendous promise for patients with rare diseases, offering new treatment options and potentially improving their lives.

## Author contributions

LC: Data curation, Writing – original draft. VM: Software, Writing – review & editing. ST: Writing – review & editing. JB: Writing – review & editing. OB: Writing – review & editing.

## Footnotes

^1^ https://www.ncbi.nlm.nih.gov/pmc/articles/PMC7347027/

^2^ https://link-springer-com.proxy.insermbiblio.inist.fr/article/10.1007/s11042-022-13428-4

^3^ https://everycure.org/

^4^ https://repo4.eu/

^5^ https://www.opentargets.org/

^6^ https://repo-hub.broadinstitute.org/repurposing#home

^7^ https://remedi4all.org/

^8^ https://healx.ai/

^9^ https://www.biovista.com/
